# Immunostaining for Homer reveals the majority of excitatory synapses in laminae I–III of the mouse spinal dorsal horn

**DOI:** 10.1016/j.neuroscience.2016.05.009

**Published:** 2016-08-04

**Authors:** Maria Gutierrez-Mecinas, Emily D. Kuehn, Victoria E. Abraira, Erika Polgár, Masahiko Watanabe, Andrew J. Todd

**Affiliations:** a Institute of Neuroscience and Psychology, College of Medical, Veterinary and Life Sciences, University of Glasgow, Glasgow G12 8QQ, UK; b Department of Anatomy, Hokkaido University School of Medicine, Sapporo 060-8638, Japan; c Department of Neurobiology, Howard Hughes Medical Institute, Harvard Medical School, Boston, MA 02115, USA

**Keywords:** CGRP, calcitonin gene-related peptide, DAB, diaminobenzidine, IB4, isolectin B4, LTMR, low-threshold mechanoreceptor, VGAT, vesicular GABA transporter, VGLUT1, vesicular glutamate transporter 1, VGLUT2, vesicular glutamate transporter 2, glutamatergic synapse, confocal microscopy, spinal cord, pain, primary afferent, excitatory interneuron

## Abstract

The spinal dorsal horn processes somatosensory information before conveying it to the brain. The neuronal organization of the dorsal horn is still poorly understood, although recent studies have defined several distinct populations among the interneurons, which account for most of its constituent neurons. All primary afferents, and the great majority of neurons in laminae I–III are glutamatergic, and a major factor limiting our understanding of the synaptic circuitry has been the difficulty in identifying glutamatergic synapses with light microscopy. Although there are numerous potential targets for antibodies, these are difficult to visualize with immunocytochemistry, because of protein cross-linking following tissue fixation. Although this can be overcome by antigen retrieval methods, these lead to difficulty in detecting other antigens. The aim of this study was to test whether the postsynaptic protein Homer can be used to reveal glutamatergic synapses in the dorsal horn. Immunostaining for Homer gave punctate labeling when viewed by confocal microscopy, and this was restricted to synapses at the ultrastructural level. We found that Homer puncta were colocalized with the AMPA receptor GluR2 subunit, but not with the inhibitory synapse-associated protein gephyrin. We also examined several populations of glutamatergic axons and found that most boutons were in contact with at least one Homer punctum. These results suggest that Homer antibodies can be used to reveal the great majority of glutamatergic synapses without antigen retrieval. This will be of considerable value in tracing synaptic circuits, and also in investigating plasticity of glutamatergic synapses in pain states.

## Highlights

• Identifying glutamatergic synapses is important for tracing synaptic circuits.
• Most proteins at glutamatergic synapses are masked by tissue fixation.
• Homer can reveal glutamatergic synapses without the need for antigen retrieval.

## Introduction

The dorsal horn of the spinal cord is innervated by primary afferents that terminate in a highly ordered lamina-specific pattern (Todd, 2010, Abraira and Ginty, 2013, Braz et al., 2014). These contribute to complex synaptic circuits that involve spinal projection neurons, local interneurons and axons that descend from the brain. The incoming sensory information undergoes extensive processing and modulation, before being transmitted to the brain via the projection neurons, where it contributes to conscious perception.

Despite the importance of the dorsal horn in pain mechanisms, the organization of its synaptic circuitry is still poorly understood (Graham et al., 2007, Todd, 2010). The vast majority of neurons in laminae I–III are interneurons, and these can be broadly divided into excitatory (glutamatergic) and inhibitory (GABAergic/glycinergic) populations. All primary afferents and some descending axons use glutamate as their principal fast transmitter, and glutamatergic synapses in the dorsal horn can therefore originate from a variety of sources.

An important advance in our understanding of the organization of neuronal circuitry has come from the ability to define different classes of glutamatergic axons. For example, unmyelinated nociceptive primary afferents can be assigned to two major classes: peptidergic and non-peptidergic, based on expression of calcitonin-gene related peptide (CGRP) and binding of the lectin IB4 from *Bandeiraea simplicifolia*, respectively (Snider and McMahon, 1998, Braz et al., 2014). Similarly, different types of low-threshold mechanoreceptive (LTMR) afferent can be recognized by their dependence on neurotrophic factor receptors and their expression of vesicular glutamate transporters (Todd et al., 2003, Alvarez et al., 2004, Brumovsky et al., 2007, Luo et al., 2009, Seal et al., 2009). The axons of local excitatory interneurons contain high levels of the vesicular glutamate transporter 2 (VGLUT2) (Todd et al., 2003, Yasaka et al., 2010), and recent studies have identified non-overlapping populations among these neurons, based on expression of various neuropeptides (Gutierrez-Mecinas et al., 2014, Gutierrez-Mecinas et al., 2016). It is therefore possible to identify several different types of glutamatergic axon with immunocytochemistry, and this should allow their location within the synaptic circuitry of the dorsal horn to be defined. However, although confocal microscopy combined with multiple-labeling immunofluorescence staining can reveal contacts between specific types of glutamatergic axons and identified dorsal horn neurons, these are not necessarily associated with synapses (Spike et al., 2002), and it is not usually possible to confirm that excitatory synapses are present at sites of contact. We have developed a method for achieving this by combining multiple-labeling immunofluorescence and confocal microscopy with subsequent electron microscopy on the same tissue (Naim et al., 1997, Baseer et al., 2012, Ganley et al., 2015). However, this is very labor-intensive, as each contact has to be identified with the electron microscope to confirm the presence of a synaptic specialization.

Confocal microscopy can be used to define circuits involving GABAergic or glycinergic inhibitory synapses, because the postsynaptic protein gephyrin, which is associated with inhibitory synapses (Fritschy et al., 2008), can be readily detected in perfusion-fixed tissue. We have used this approach to demonstrate selective innervation of dorsal horn neurons by specific populations of inhibitory interneurons (Polgár et al., 2011, Ganley et al., 2015). Although there are numerous proteins in the postsynaptic density of glutamatergic synapses (e.g. ionotropic glutamate receptor subunits and PSD-95), the antigenic epitopes on these proteins are either embedded within the postsynaptic density or in the synaptic cleft, both of which have a highly complex structure (Sheng and Hoogenraad, 2007, Ryan and Grant, 2009). The extensive protein cross-linking within these regions that results from formaldehyde fixation (Fox et al., 1985) therefore makes these epitopes difficult to detect with conventional immunocytochemistry in fixed tissue (Ottersen and Landsend, 1997, Fritschy et al., 1998, Watanabe et al., 1998). These proteins can be revealed by antigen retrieval, for example by treatment with pepsin (Watanabe et al., 1998, Fukaya and Watanabe, 2000, Nagy et al., 2004a, Nagy et al., 2004b, Polgár et al., 2008), but we have found that this disrupts many other antigens, making it difficult to define the pre- and postsynaptic elements. Antibodies against the postsynaptic scaffolding protein Homer have been used elsewhere in the CNS to reveal glutamatergic synapses (Dani et al., 2010), and the aim of this study was to determine their suitability for detecting these synapses in laminae I–III of the spinal dorsal horn.

## Experimental procedures

All experiments were approved by the Ethical Review Process Applications Panel of the University of Glasgow, and were performed in accordance with the European Community directive 86/609/EC and the UK Animals (Scientific Procedures) Act 1986. Efforts were made to minimize the number of animals used and their suffering.

### Distribution of Homer and its association with glutamatergic boutons

Four adult C57Bl/6 mice of either sex (20–27 g) were deeply anesthetized with pentobarbitone (30 mg i.p.) and perfused through the left cardiac ventricle with fixative consisting of 4% freshly depolymerized formaldehyde in phosphate buffer. Midlumbar segments (L3-5) were removed and stored at 4 °C for 2 h in the same fixative, before being cut into 60 μm thick sections with a vibrating blade microtome. The sections were immersed for 30 min in 50% ethanol to enhance antibody penetration. In some cases, binding of isolectin B4 (IB4) from *Bandeiraea simplicifolia* (Wang et al., 1994, Sakamoto et al., 1999) was used to identify C fibers, and in these cases the sections were incubated overnight in IB4 (1 μg/ml; Vector Laboratories, Peterborough, UK). Sections were reacted for multiple-labeling immunofluorescence staining as described previously (Gutierrez-Mecinas et al., 2014, Gutierrez-Mecinas et al., 2016, Cameron et al., 2015, Ganley et al., 2015). Details of the antibodies used in this study, including the sources and concentrations, are provided in Table 1. The sections were incubated for 3 days at 4 °C in primary antibodies, as we have found that penetration of immunostaining in fine axonal processes is often improved by this prolonged incubation. The antibodies were diluted in PBS that contained 0.3 M NaCl, 0.3% Triton X-100 and 5% normal donkey serum. They were then incubated overnight in species-specific secondary antibodies that were raised in donkey and conjugated to Alexa 488, Alexa 647, Rhodamine Red or biotin (Jackson Immunoresearch, West Grove, PA, USA). All secondary antibodies were diluted 1:500 (in the same diluent), apart from those conjugated to Rhodamine Red, which were diluted 1:100. Biotinylated secondary antibodies were detected with Pacific Blue conjugated to avidin (1:1000; Life Technologies, Paisley, UK). Sections from 2 animals were reacted with each of the following combinations of primary antibodies: (1) Homer, CGRP and VGLUT2 (goat antibody), (2) Homer, vesicular glutamate transporter 1 (VGLUT1) and VGLUT2 (guinea-pig antibody), (3) Homer, gephyrin, vesicular GABA transporter (VGAT) and VGLUT2 (guinea-pig antibody). Those that had been pre-incubated with IB4 were reacted with antibodies against Homer, IB4 and CGRP. Sections were mounted in anti-fade medium and stored at −20 °C.

The sections were scanned with a Zeiss LSM710 confocal microscope equipped with Argon multi-line, 405 nm diode, 561 nm solid state and 633 nm HeNe lasers, and a spectral detection system. In most cases, confocal image stacks (z-separation of 0.3 μm) were obtained through a 63× oil-immersion lens (numerical aperture 1.4) with the aperture set to 1 Airy unit or less. The resulting z-stacks were analyzed with Neurolucida for Confocal software (MBF Bioscience, Williston, VT, USA). In most of the immunoreacted sections, we did not have suitable markers to identify laminar boundaries, and we therefore defined laminae I, II and III as parallel bands that were 20 μm, 60 μm and 80 μm thick, respectively, measured from the dorsalmost part of the dorsal horn (Polgar et al., 2013, Ganley et al., 2015).

Boutons belonging to non-peptidergic C fibers were identified by the presence of IB4, but since IB4 also binds to some peptidergic afferents (most of which express CGRP) (Sakamoto et al., 1999), only IB4-labeled boutons that lacked CGRP (IB4^+^/CGRP^−^) were included in this part of the analysis. Immunostaining for CGRP was used to reveal central terminals of peptidergic boutons. We found that these also showed weak VGLUT2-immunoreactivity, and since this allowed the boutons to be distinguished from intervaricose portions of peptidergic primary afferents, we selected peptidergic boutons based on colocalization of CGRP and VGLUT2 (CGRP^+^/VGLUT2^+^). All myelinated LTMR (A-LTMR) cutaneous afferents are thought to express VGLUT1 (Oliveira et al., 2003, Todd et al., 2003, Alvarez et al., 2004), and these arborize in the inner half of lamina II (lamina IIi) and throughout laminae III–V (Hughes et al., 2003, Abraira and Ginty, 2013). Although corticospinal axons also express VGLUT1 and terminate in the dorsal horn, it is likely that the majority of VGLUT1^+^ boutons in lamina IIi and III belong to A-LTMRs, and we therefore analyzed VGLUT1^+^ boutons in this region.

Since glutamatergic neurons in the dorsal horn express VGLUT2 (Yasaka et al., 2010), which is present at a high level in their axonal boutons (Todd et al., 2003), we used the presence of strong VGLUT2 immunoreactivity to identify the axons of putative local glutamatergic neurons, and this analysis was performed on sections reacted for Homer, VGLUT2 and CGRP.

To analyze the association of Homer puncta with different types of glutamatergic bouton, we selected the boutons while the channel corresponding to Homer was switched off, and ensured that the selected boutons were distributed throughout the dorsoventral extent of the region being analyzed. When the selection was complete, we switched on the Homer channel and quantified the proportion of the selected boutons that were in contact with at least one Homer punctum (i.e. with no intervening pixels) by following the bouton through its rostrocaudal length in the confocal z-series.

Peptidergic boutons terminate in a plexus that occupies laminae I and IIo, and we therefore sampled CGRP^+^ boutons throughout this region. Non-peptidergic nociceptors, identified by IB4-binding and lack of CGRP have a narrow termination zone in the mid-part of lamina II, and this region was used to select IB4^+^/CGRP^−^ boutons. Myelinated low-threshold afferents arborize throughout lamina IIi-V, and it is known that different classes have specific laminar termination zones (Abraira and Ginty, 2013). We therefore sampled VGLUT1^+^ boutons in laminae IIi and III separately. Boutons with strong VGLUT2-immunoreactivity, which are likely to be derived mainly from local excitatory interneurons, are found throughout the dorsal horn (Todd et al., 2003, Alvarez et al., 2004). These are thought to originate from several distinct populations of interneurons, which have axons that arborize in different laminar locations (Gutierrez-Mecinas et al., 2016), and so we analyzed VGLUT2^+^ boutons in laminae I, II and III separately. In all cases, 100 neurochemically defined boutons were analyzed in each of 2 mice, except for IB4^+^/CGRP^−^ boutons, which are relatively less numerous than the other types, and for which 50 boutons were analyzed per mouse.

### Association of Homer with GluR2 subunit of the AMPA receptor

To investigate the relationship between Homer and GluR2, we processed sections from two of the mice with an antigen retrieval method that can reveal ionotropic receptors at glutamatergic synapses (Watanabe et al., 1998, Nagy et al., 2004a, Polgár et al., 2008). This was necessary, because GluR2 is not normally detectable at synapses in perfusion fixed spinal cord tissue without antigen retrieval (Nagy et al., 2004a). Sections were incubated for 30 min in PBS at 37 °C, followed by 10 min in 0.2 M HCl containing 0.25 mg/ml pepsin (Dako, Glostrup, Denmark). They were then rinsed and reacted with antibodies against Homer and GluR2, which were revealed with fluorescent secondary antibodies as described above. Sections were scanned with the confocal microscope and analyzed with Neurolucida for Confocal.

### Ultrastructural distribution of Homer

In order to confirm that the staining seen with Homer antibody was located in postsynaptic densities, we examined tissue from two adult male NIHS mice (31 or 32 g) that had been used in a previous study (Iwagaki et al., 2013), and this was processed by a pre-embedding immunoperoxidase method (Polgár and Todd, 2008). The mice had been perfused with fixative that contained 0.2% glutaraldehdye/4% formaldehyde, and transverse sections of the L3 segment were treated with 50% ethanol for 30 min to enhance antibody penetration, followed by 30 min in 1% sodium borohydride to reduce free aldehyde groups. They were incubated overnight in Homer antibody (diluted 1:20,000 in PBS) and then in biotinylated donkey anti-rabbit antibody, followed by avidin conjugated to horseradish peroxidase. The sections were then reacted with 3,3’-diaminobenzidine (DAB), osmicated (1% OsO4 for 20 min), dehydrated in acetone, block stained with uranyl acetate and flat-embedded in Durcupan. Ultrathin sections were cut with a diamond knife, collected on Formvar-coated slot grids and stained with lead citrate. They were viewed on a Philips CM100 electron microscope.

### Characterization of antibodies

The affinity-purified Homer antibody was raised against amino acids 1–175 of mouse Homer 1 and detects a band at 43–45 kDa in immunoblots of mouse brain extracts (Nakamura et al., 2004). Since the first 120 amino acids are highly conserved between Homer 1, 2 and 3 the antibody is likely to detect all forms of Homer. The IB4 antibody was raised against the lectin from *Bandeiraea simplicifolia* and specificity is shown by the lack of staining in tissue that does not contain the lectin. The antibody against CGRP detects both α and β forms of the peptide (manufacturer’s specification). The guinea-pig and goat antibodies against VGLUT2 were raised against peptides corresponding to amino acids 565-582 of rat VGLUT2 (guinea-pig antibody) and amino acids 550-582 of mouse VGLUT2 (goat antibody). The guinea-pig antibody stains identical structures to a well-characterize rabbit VGLUT2 antibody (Todd et al., 2003), and the goat antibody detects a single protein band of the appropriate molecular weight (60 kDa) (Kawamura et al., 2006). The goat anti-VGLUT1 and anti-VGAT antibodies were raised against amino acids 531-560 of mouse VGLUT1 and amino acids 31-112 of mouse VGAT, and both label bands of the appropriate size on Western blots (Kawamura et al., 2006, Miura et al., 2006). The gephyrin antibody was generated against an extract of rat spinal cord synaptic membranes (Pfeiffer et al., 1984). It has been extensively characterized and shown to bind to a 93-kDa peripheral membrane protein (gephyrin) in extracts of rat brain membranes (Becker et al., 1989). The monoclonal GluR2 antibody (clone 6C4) has been extensively characterized and shown not to detect other AMPA or kainate subunits (Vissavajjhala et al., 1996).

## Results

### Distribution of Homer at the light and electron microscopic levels

**Fig. 1:**
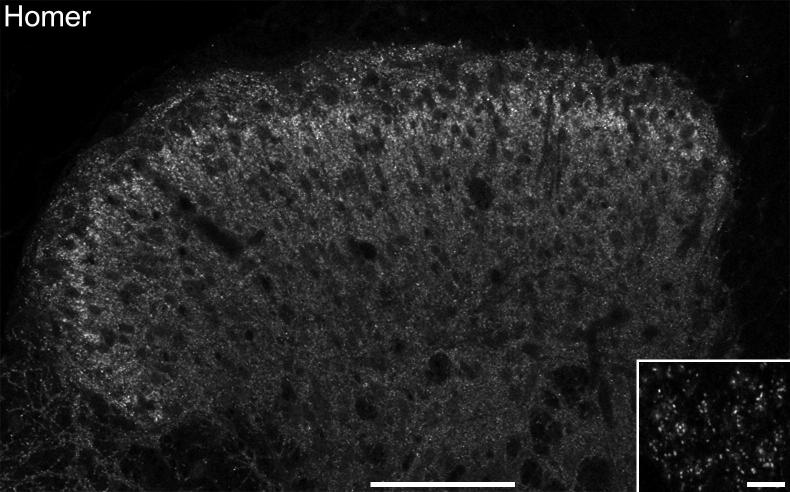
A low magnification view of Homer-immunoreactivity in a transverse section through the dorsal horn. Immunostaining is present throughout the gray matter, but is densest in lamina II. The inset is a higher magnification view of part of lamina II, and shows that the staining is in the form of small puncta that are scattered throughout the neuropil. Both images are from a single optical section. Scale bars: 100 μm (main image) and 5 μm (inset).

Immunostaining with the Homer antibody appeared as small puncta of varying size and intensity. These were present throughout the spinal gray matter, but were densest in lamina II (Fig 1). This distribution resembled that seen with antibodies against the GluR2 subunit of the AMPAr or PSD-95 following antigen retrieval with pepsin (Nagy et al., 2004a, Polgár et al., 2008), however, Homer could be readily detected without pepsin treatment. We have also obtained immunostaining with an apparently identical distribution, using a rabbit antibody from a different source (Synaptic Systems, catalog number 160003).

**Fig. 2:**
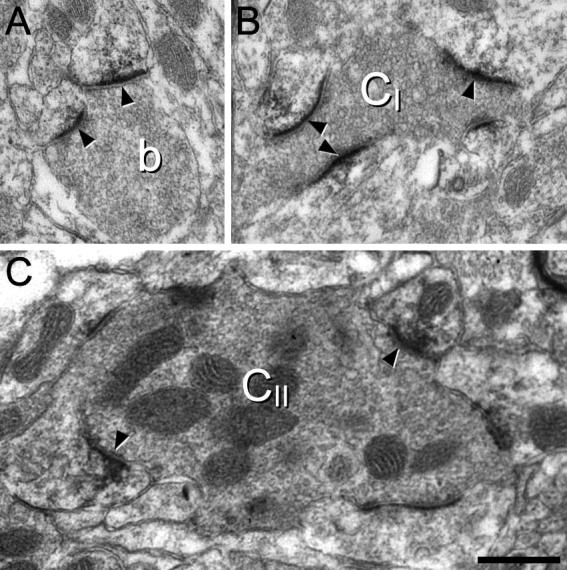
The ultrastructural appearance of Homer, seen with pre-embedding immunoperoxidase labeling. The DAB reaction product is confined to synapses, where it is always restricted to the postsynaptic aspect. (A) a bouton (b) makes two synapses (arrowheads), both of which show DAB labeling. (B) The central bouton of a type I glomerulus (C_I_) can be recognized because of its indented contour, lack of mitochondria and the presence of densely packed synaptic vesicles of highly variable size. Three of the synapses (arrowheads) formed by the central bouton are strongly labeled with DAB. (C) The central bouton of a type II glomerulus (C_II_) can be identified because of its large size and numerous mitochondria. It forms several synapses with adjacent peripheral profiles, and two of these (arrowheads) are clearly DAB-labeled. Scale bar = 0.5 μm.

With electron microscopy, the DAB precipitate was only detected at synapses, where it was invariably located on the post-synaptic aspect (Fig. 2). It was not possible to determine whether these were asymmetrical or symmetrical, because the DAB obscured the appearance of the postsynaptic density. The DAB reaction product could generally be distinguished from unlabeled postsynaptic densities, because it extended into the underlying cytoplasm, giving the postsynaptic density a ragged appearance. Immunostaining was detected postsynaptic to boutons that formed only one or two synapses in the plane of section (Fig 2a), as well as to boutons that formed the central component of type I and type II synaptic glomeruli (Ribeiro-da-Silva and Coimbra, 1982). As reported previously, the central boutons of type I glomeruli were small, indented and relatively dark, with few mitochondria and densely packed synaptic vesicles of variable diameter (Fig. 2b). In contrast, central boutons of type II glomeruli were typically larger, with numerous mitochondria and less densely packed synaptic vesicles (Fig. 2c). DAB was not detected at all asymmetrical synapses, and this could be due to lack of penetration of antibodies in the absence of detergent, suppression of immunostaining by glutaraldehyde in the fixative, and the difficulty of distinguishing weak DAB label that was restricted to the postsynaptic density.

### Relation of Homer to other postsynaptic proteins

**Fig. 3:**
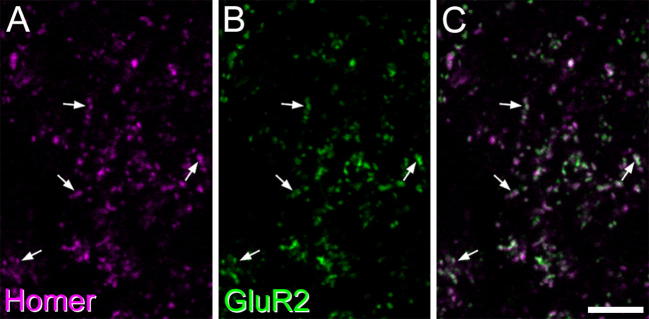
A confocal scan (single optical section) through the middle part of lamina II in a section that had been pepsin-treated and then immunostained with antibodies against Homer and the AMPA receptor GluR2 subunit. (A, B) Homer- and GluR2-immunoreactive puncta are shown in magenta and green, respectively. (C) A merged image. Note that numerous puncta are present (some indicated with arrowheads) and that virtually all of these are stained with both antibodies, although the relative intensity of staining with the two antibodies varies considerably. Scale bar = 5 μm.

The GluR2 subunit of the AMPA receptor is thought to be present at virtually all excitatory synapses in laminae I–III of the dorsal horn. This assumption is based on studies of rat dorsal horn involving antigen retrieval with pepsin, in which we found that 99% of puncta that were labeled with an antibody that recognizes all 4 subunits of the AMPA receptor (pan-AMPAr antibody) were also GluR2-immunoreactive, and that 98% of puncta labeled with antibody against the major postsynaptic density protein PSD-95 were also pan-AMPAr-immunoreactive (Polgár et al., 2008). We therefore compared the distribution of Homer and GluR2 in the superficial dorsal horn of the mouse. Since synaptic AMPAr subunits cannot generally be detected without antigen retrieval (Nagy et al., 2004a), we used sections that had been treated with pepsin and found that Homer could still be detected, with a similar distribution to that seen without pepsin treatment. The results of the quantitative analysis of puncta in laminae I–III are shown in Table 2, and a typical example is illustrated in Fig 3. When results across the 3 laminae were pooled, 94% of Homer puncta were also GluR2-immunoreactive, while 97% of GluR2 puncta were Homer-immunoreactive. The lack of GluR2 at some Homer puncta is likely to have resulted from the weaker pepsin treatment in this study (0.25 mg/ml, compared to 1 mg/ml in Nagy et al., 2004a), which was used to restrict any loss of Homer staining that might result from pepsin digestion.

**Fig. 4:**
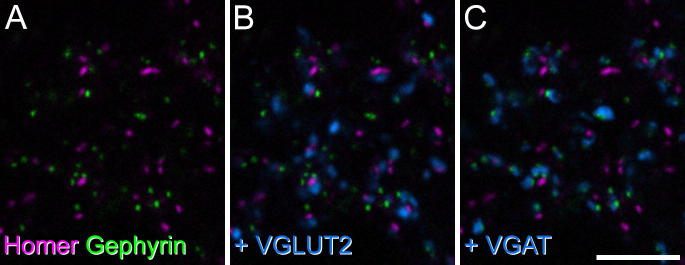
Confocal scan (single optical section) of a section reacted with antibodies against Homer, gephyrin, VGLUT2 and VGAT. (A) Numerous puncta immunoreactive for Homer (magenta) and gephyrin (green) are present in the neuropil of lamina II, but these are never co-localized. (B) The same field scanned to reveal VGLUT2 (blue) shows that some of the Homer puncta are in contact with VGLUT2-immunoreactive boutons. (C) The same field showing staining for VGAT (blue) reveals that most of the gephyrin puncta are adjacent to VGAT-immunoreactive boutons. Scale bar = 5 μm.

We have previously provided evidence that gephyrin can be detected at the great majority of inhibitory synapses in laminae I–III, since there is a close association between gephyrin puncta and boutons that contain VGAT (Sardella et al., 2011), which is present in all GABAergic and glycinergic terminals (Chaudhry et al., 1998). We therefore compared the distribution of the two proteins in sections that were also immunostained for VGAT and VGLUT2 (Fig. 4). We found that although numerous gephyrin- and Homer-immunoreactive puncta were intermingled throughout the dorsal horn, these were never co-localized (Fig 4a). As expected, the gephyrin puncta were in contact with VGAT^+^ boutons, while in many cases the Homer puncta were in contact with VGLUT2^+^ boutons (Fig 4b, c).

### Association with different classes of glutamatergic axonal bouton

**Fig. 5:**
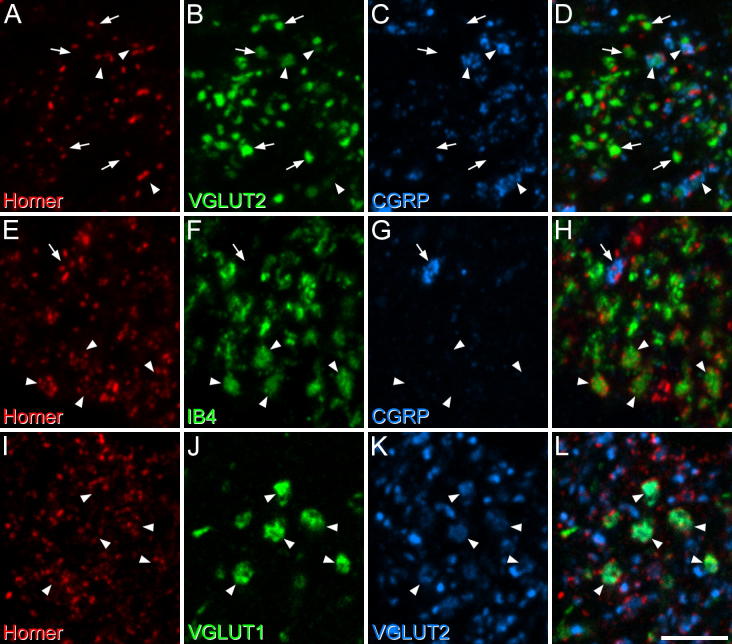
Confocal images showing the association between Homer puncta and various types of glutamatergic axon in laminae I–III of the dorsal horn. (A–D) part of lamina I from a section stained to reveal Homer (red), VGLUT2 (green) and CGRP (blue). This field contains a few CGRP-immunoreactive boutons (arrowheads), which are also weakly stained for VGLUT2, and numerous boutons that lack CGRP and show strong VGLUT2 immunoreactivity (some indicated with arrows). The merged image shows that the CGRP-immunoreactive boutons, and many of those with strong VGLUT2, are in contact with Homer puncta. Note that some of the boutons may be in contact with Homer puncta that are above or below the z-stack, and would therefore not be seen in this image. (E–H) A similar set of confocal images from lamina II in a section stained to reveal Homer (red), IB4 binding (green) and CGRP (blue). Although much of the IB4 is bound to intervaricose axons (as judged from the confocal z-stack), some of the labeled profiles are boutons (arrowheads), and each of these is surrounded by numerous Homer puncta. A single CGRP bouton is present (arrow) and this is also in contact with Homer puncta. (I–L) A similar set of confocal images from lamina III in a section stained to reveal Homer (red), VGLUT1 (green) and VGLUT2 (blue). Several large VGLUT1-immunoreactive boutons are visible (some indicated with arrowheads). These show weak VGLUT2 immunoreactivity, and each is associated with numerous Homer puncta. In addition, the field contains a large number of boutons that lack VGLUT1 but show strong VGLUT2 immunoreactivity, and many of these are in contact with Homer puncta. The images were generated from 5 (A–D), 4 (E–H) and 3 (I–L) confocal optical sections at 0.3 μm z-spacing. Scale bar = 5 μm.

CGRP^+^ boutons, which showed weak VGLUT2-immunoreactivity, were found at highest density in laminae I and IIo, but were also scattered in the deeper laminae, a distribution which resembles that seen in the rat. We found that virtually all of these (mean 98.5%) were in contact with at least one Homer punctum (Fig. 5a–d; Table 3).

As in the rat, IB4-binding revealed a dense plexus of axons in the mid-part of lamina II, with occasional profiles superficial or deep to this. Although some IB4^+^ profiles were also CGRP-immunoreactive, the great majority lacked CGRP, and these included both intervaricose portions of axons and relatively large varicosities. These could be distinguished in confocal z-stacks, because of rapid change in size of the boutons across a limited number of z-sections. All of the IB4^+^/CGRP^−^ varicosities analyzed were associated with Homer puncta (Table 3), and in most cases several puncta surrounded the varicosity (Fig. 5e–h). This arrangement presumably corresponds to the expression of Homer in dendrites postsynaptic to central endings of type I glomeruli (Fig 2b), which originate from the non-peptidergic C nociceptors that are labeled with IB4 (Ribeiro-da-Silva and Coimbra, 1982, Ribeiro-da-Silva and Coimbra, 1984, Gerke and Plenderleith, 2004).

VGLUT1-immunoreactive boutons showed a similar distribution to that reported in rat, with sparse labeling in laminae I–IIo, and a dense plexus that extended from the mid-part of lamina II through the remainder of the dorsal horn. Many of these profiles also showed weak labeling for VGLUT2 (Fig 5i–l), as reported in the rat (Todd et al., 2003). Virtually all (99.8%) of the VGLUT1-immunoreactive boutons analyzed in laminae IIi and III were in contact with Homer puncta (Table 3). As for the IB4-labeled profiles in mid-lamina II, the VGLUT1-immunoreactive boutons were generally in contact with more than 1 Homer punctum, and in many cases they were surrounded by these puncta (Fig 5i–l). These presumably correspond to type II glomeruli (Fig 2c), which are centered around A-LTMR afferents (Ribeiro-da-Silva and Coimbra, 1982).

VGLUT2-immunoreactive boutons were present throughout the dorsal horn, and these were analyzed in sections that had been stained with antibodies against VGLUT2, CGRP and Homer. VGLUT2 is expressed in many IB4-labeled and VGLUT1-immunoreactive boutons in this region (see above), but these generally show a relatively low level of VGLUT2-immunoreactivity (e.g. Fig 5k). In contrast, there are large numbers of boutons with very strong VGLUT2-immunoreactivity, and these are likely to correspond largely to the axons of local glutamatergic interneurons (Todd et al., 2003). We therefore analyzed boutons that showed strong VGLUT2 (and lacked CGRP) in each of laminae I, II and III. Most of these (84.5–92%, Table 3) were in contact with Homer puncta, but unlike the VGLUT1- and IB4-labeled boutons, they were generally contacted by only one or two puncta, and were never surrounded by them (Figs 5a–d, i–l). When data from the 3 laminae were pooled, 88.8% of the VGLUT2 boutons were contacted by Homer puncta.

## Discussion

The main findings of this study are: (1) that Homer can be detected in the spinal dorsal horn without the need for antigen retrieval, (2) that the resulting punctate staining is apparently restricted to excitatory synapses, and (3) that the great majority of glutamatergic boutons identified with VGLUT1, VGLUT2, CGRP or IB4 binding are in contact with at least one Homer punctum.

### Homer expression at glutamatergic synapses

A short form of the Homer protein (now known as Homer1a) was initially identified as an immediate early gene that was induced by neuronal activation (Brakeman et al., 1997, Kato et al., 1997). Subsequent investigations revealed a family of closely related proteins derived from 3 different genes (*Homer1*, *Homer2*, *Homer3*), each of which gave rise to several splice variants (Kato et al., 1998, Xiao et al., 1998, Shiraishi-Yamaguchi and Furuichi, 2007). The proteins belonging to the 3 Homer families are differentially distributed within the nervous system, with Homer3 being largely restricted to the cerebellum and hippocampus, and Homer1 and 2 being more widely expressed throughout the CNS (Shiraishi-Yamaguchi and Furuichi, 2007, Tao-Cheng et al., 2014). All of the Homer proteins possess a highly conserved N-terminal (EVH1-like) domain, while the long forms (Homer1b, 1c, 2a, 2b, 3a, 3b) have a C-terminal coiled-coil domain that includes two leucine zipper motifs, which allow homomeric or heteromeric protein interactions. The long forms are constitutively expressed and concentrated in the postsynaptic density of glutamatergic synapses, where they can interact with a variety of proteins, including Shank and metabotropic glutamate receptors (mGluRs), and are thought to have an important role in Ca^2+^ signaling (Tu et al., 1999, Shiraishi-Yamaguchi and Furuichi, 2007, Worley et al., 2007, Foa and Gasperini, 2009). In contrast, the short forms (Homer 1a, 2c, 2d, 3c, 3d), which are normally expressed at low levels and are induced by neuronal activation, are thought to disrupt the normal actions of the long forms of Homer by competing for their binding partners (Shiraishi-Yamaguchi and Furuichi, 2007, Foa and Gasperini, 2009).

Several anatomical studies have demonstrated the distribution of Homer within postsynaptic densities of glutamatergic synapses with electron microscopy (Xiao et al., 1998, Tu et al., 1999, Petralia et al., 2001, Petralia et al., 2005, Tao-Cheng et al., 2014) or light microscopic methods (Dani et al., 2010, Nair et al., 2013, Andreska et al., 2014). Dani et al. (2010) examined the precise synaptic localization of Homer1 with super-resolution microscopy and found that in the plane of the synapse, the Homer1 puncta were co-extensive with those immunostained by Bassoon, a component of the presynaptic active zone. This supports electron microscopic evidence that Homer is present throughout the postsynaptic density, but does not extend into the perisynaptic zone (Tao-Cheng et al., 2014). In addition, Dani et al. estimated the distance of several proteins from the synaptic cleft in the axial plane, and concluded that Homer1 was located further from the cleft (∼80 nm) than the other proteins examined, which included NR2B and GluR1 subunits, PSD-95, Shank and CaMKII.

We have previously reported that although various subunits of the NMDA and AMPA receptors (Nagy et al., 2004a, Nagy et al., 2004b, Polgár et al., 2008), as well as PSD-95 (Polgár et al., 2008) can be detected at glutamatergic synapses in the dorsal horn with immunocytochemistry, these required antigen retrieval with pepsin, presumably because the epitopes were masked by protein cross-linking in the postsynaptic density or synaptic cleft as a result of aldehyde fixation (Watanabe et al., 1998). The finding that Homer can be readily detected without antigen retrieval (even in glutaraldehyde-fixed tissue) is presumably because the relevant epitopes are much more superficially located within the postsynaptic density (i.e. further from the synaptic cleft), such that they are accessible to antibodies.

### Do all glutamatergic synapses in laminae I–III contain Homer?

Our results suggest that Homer was restricted to the postsynaptic density of glutamatergic synapses, but an important question is whether all of these synapses could be detected with the Homer antibody. Although the antibody was raised against Homer1, it is likely to recognize all 3 forms of the protein. We previously presented evidence that following pepsin treatment, virtually all glutamatergic synapses in laminae I–III of the rat dorsal horn could be revealed with the monoclonal antibody against GluR2 that was used here. In the present study, we found that after pepsin treatment 97% of GluR2 puncta were Homer-immunoreactive, which would be consistent with the view that the great majority of glutamatergic synapses were labeled with the Homer antibody. However, although virtually all of the boutons labeled with CGRP, IB4 and VGLUT1 were associated with at least one Homer punctum (and in many cases, were surrounded by puncta), we consistently found that ∼10–15% of VGLUT2 boutons were not in contact with a Homer punctum. Primary afferent boutons frequently form multiple synapses, and in many cases glomerular arrangements (Ribeiro-da-Silva and Coimbra, 1982), whereas boutons belonging to excitatory interneurons appear to form only 1 or 2 synapses (Nagy et al., 2004a), and this is likely to account for the very high proportion of CGRP, IB4 and VGLUT1 boutons that contacted Homer puncta.

However, there is a discrepancy between the present results and our previous findings in the rat, because we reported that ∼95% of VGLUT2 boutons in laminae I–III of the rat were in contact with at least one GluR2 punctum, whereas here we find that ∼88% of these boutons have an adjacent Homer punctum. This could reflect a species difference (e.g. if slightly fewer VGLUT2 boutons form synapses in the mouse), but it may also be that we failed to detect Homer at some glutamatergic synapses. Although Homer is clearly detectable without antigen retrieval, it is possible that pepsin treatment would have resulted in immunostaining at some synapses that had very low levels of the protein.

It remains to be seen whether the few VGLUT2 boutons that lack Homer puncta form synapses, but in any case, it is likely that the great majority of glutamatergic synapses are labeled with the Homer antibody. This should provide an extremely useful way of identifying excitatory synapses between different types of neuron in the dorsal horn in anatomical studies, complementing other approaches, including those based on use of transgenic mouse lines (Borgius et al., 2010).

### Roles of Homer in spinal pain mechanisms

Several studies have investigated the role of Homer in neuropathic and inflammatory pain models in both rats and mice. It has been reported that levels of Homer1b/c in the postsynaptic density fraction show an early reduction and then a prolonged increase in the dorsal horn ipsilateral to a chronic constriction (neuropathic) injury (Miletic et al., 2005, Miletic et al., 2009, Obara et al., 2013), and a similar prolonged increase was seen following inflammation induced by an intra-articular injection of complete Freund’s adjuvant (Yao et al., 2011). It has also been shown that disrupting the function of Homer1b/c with intrathecally administered antisense oligonucleotides led to a reduction of pain in an inflammatory model (Yao et al., 2011, Yao et al., 2014), while over-expression of Homer1c/2b in the dorsal horn exacerbated pain in the chronic constriction injury model (Obara et al., 2013). mRNA for Homer1a increases rapidly in the ipsilateral dorsal horn in both inflammatory and neuropathic models (Miyabe et al., 2006, Tappe et al., 2006), and it has been proposed that this exerts negative feedback to limit pain, by competing with the long forms of Homer, and therefore uncoupling glutamate receptors at the synapse from intracellular signaling pathways (Miyabe et al., 2006, Tappe et al., 2006, Obara et al., 2013).

The increased expression of the long forms of Homer (which are located within the postsynaptic density) in different pain models could reflect an increased density of the protein in glutamatergic synapses that are otherwise unchanged, but it may also result from an increase in the number and/or size of these synapses. Since we have shown that Homer can be readily detected in individual synapses with immunocytochemistry and confocal microscopy, it should be possible to distinguish between these possibilities by performing a quantitative analysis of Homer immunoreactivity in the dorsal horn in chronic pain models and assessing the size and frequency of Homer puncta. Any changes observed could also be related to the different types of glutamatergic axon that give rise to these synapses, as well as to the dorsal horn neurons that are postsynaptic.

## Conclusion

These results demonstrate that antibody against Homer can be used to reveal glutamatergic synapses in laminae I–III of the dorsal horn for confocal microscopy, without the need for antigen retrieval. This will be important for studies of neuronal circuits that underlie pain processing in the spinal cord, and also for future studies designed to investigate plasticity in excitatory synapses in chronic pain states.

## Figures and Tables

**Table 1:** Antibodies used in this study

Antibody	Species	Catalog no	Dilution	Source
Homer	Rabbit	Homer1-Rb-Af1000	1:2000 1:20,000^⁎^	Frontier Science
IB4	Goat	AS-2104	1:2000	Vector Laboratories
CGRP	Guinea pig	T-5027	1:5000	Peninsula
VGLUT2	Goat		1:500	M Watanabe
VGLUT2	Guinea pig	ab2251	1:2000–5000	Millipore
VGLUT1	Goat		1:500	M Watanabe
VGAT	Goat		1:1000	M Watanabe
Gephyrin	Mouse	147 021	1:2000	Synaptic Systems
GluR2	Mouse	MAB397	1:300	Millipore

^⁎^ For electron microscopy.

**Table 2:** The extent of co-localization of Homer with GluR2 in tissue that had undergone antigen retrieval

	% of Homer puncta with GluR2	% of GluR2 puncta with Homer
Lamina I	94.5 (93, 96)	97.5 (97, 98)
Lamina II	93.5 (93, 94)	98.5 (98, 99)
Lamina III	93.5 (91, 96)	94.5 (93, 96)
Combined	93.8	96.8

In each case the mean values for the two animals are shown, with the individual values in parentheses.

**Table 3:** Association of Homer puncta with axons of different neurochemical types

Neurochemical marker(s)	Lamina(e)	Number sampled per mouse	% associated with at least 1 Homer punctum
CGRP^+^/VGLUT2^+^	I-IIo	100	98.5 (98, 99)
IB4^+^/CGRP^−^	II	50	100 (100, 100)
VGLUT1^+^	III	100	100 (100, 100)
VGLUT1^+^	III	100	99.5 (99, 100)
VGLUT2^+^/CGRP^−^	I	100	90 (88,92)
VGLUT2^+^/CGRP^−^	II	100	84.5 (84,85)
VGLUT2^+^/CGRP^−^	III	100	92 (92,92)

In each case the mean percentages for the two animals are shown, with the individual values in parentheses.
